# Evaluation of the remineralization potential of self-assembling peptide P11-4 with fluoride compared to fluoride varnish in the management of incipient carious lesions: a randomized controlled clinical trial

**DOI:** 10.1007/s00784-024-05822-z

**Published:** 2024-07-22

**Authors:** Omar Shaalan, Karim Fawzy El-Sayed, Eman Abouauf

**Affiliations:** 1 Conservative Dentistry Department, Faculty of Dentistry, Cairo University, Al Saraya Str. 11, Manial, Cairo, Egypt; 2 Conservative Dentistry Division, School of Dentistry, Newgiza University, First 6th of October, Egypt; 3 Oral Medicine and Periodontology Department, Faculty of Dentistry, Cairo University, Giza, Egypt; 4 Clinic for Conservative Dentistry and Periodontology, School of Dental Medicine, Christian Albrechts University, Kiel, Germany

**Keywords:** Enamel, Remineralization, Non-cavitated, Incipient, Caries, Self-assembling peptide, P11-4, Fluoride, Varnish, Laser, Fluorescence

## Abstract

**Objectives:**

The present trial’s aim was to compare the remineralization potential of self-assembling peptide P11-4 combined with fluoride to that of fluoride varnish.

**Materials and methods:**

Twenty-eight participants with 58 incipient carious lesions were enrolled in the present trial. Participants were randomly divided into two groups with 14 participants and 29 incipient lesions in each group. Patients were assigned either to self-assembling peptide combined with fluoride (Curodont Repair Fluoride Plus™) or sodium fluoride varnish (NaF, Bifluorid 10) groups. Both agents were applied according to the manufacturer’s instructions on non-cavitated incipient carious lesions. Lesions were assessed by two calibrated and blinded assessors at baseline, and after one-, three- and six-months using a laser fluorescence device (DIAGNOdent).

**Results:**

Although laser fluorescence scores significantly improved in both groups over time (*p* < 0.05), no notable differences were evident between both groups at one-month (*p* > 0.05). Yet, at three- and six-months statistically lower laser fluorescence readings were evident in the self-assembling peptide combined with fluoride group in comparison to the fluoride alone group (*p* < 0.05). There was 60% less risk for caries progression for Curodont Repair Fluoride Plus™ when compared to NaF varnish after six months. Self-assembling peptide combined with fluoride was able to change 65.5% of non-cavitated carious lesions from DIAGNOdent score 3 (11–20) to score 1 (0–4). Fluoride varnish was able to change 13.8% of the lesions from score 3 to score 1 after six months.

**Conclusions:**

The self-assembling peptide combined with fluoride varnish showed higher remineralization potential than fluoride varnish alone for incipient carious lesions over a six-months follow up.

**Clinical relevance:**

The combination of self-assembling peptide P11-4 and fluoride could offer a new tool in managing incipient carious lesions.

## Introduction

Enamel is a highly mineralized tissue with an exceptional structure, high hardness, unique esthetic and physical qualities characteristics [1, 2]. Although, primarily responsible for the protection of the underlying dental structures, in case of damage it cannot self-regenerate and dental restorative interventions using artificial filling materials remain to be the solution. Yet, a possible remineralization of incipient demineralized enamel lesions using biomimetic remineralizing agents to regain the lost enamel integrity and stop the advancement of the dental decay could offer a minimally invasive early treatment option [3, 4].

Based on the biomimetic technology and guided tissue regeneration (GTR) approaches; self-assembling peptides emerged as viable alternative minimally invasive therapies and performed in a unique manner, where their protein scaffold is arranged as a matrix to induce remineralization of the initial carious lesion [5]. Although fluoride is the most common substance used to remineralize such lesions, due to its affinity and deposition on the calcium rich enamel surface, fluoride lacks the abilities for a biomimetic subsurface remineralization [6, 7]. In contrast, self-assembling peptide (SAP) was suggested as biomimetic scaffolds for enamel remineralization [8, 9]. Specifically, the self-assembling peptide P11-4 demonstrated the ability to create a three-dimensional matrix within the subsurface of early carious lesions imitating the enamel matrix proteins, templating for a hydroxyapatite nucleation [10] and remineralizing initial carious lesions [11, 12]. A combination of this self-assembling peptide with fluoride could produce synergistic effects and enhance faster and more efficient biomimetic remineralization of early carious enamel lesions [13, 14].

In this context, an early detection of carious lesions remains to be a key factor for the success of any remineralizing therapeutic protocol. Therefore, traditional methods for caries diagnosis might require additional diagnostic tools. The additional use of instruments with high sensitivity and specificity could provide accurate diagnostic tools for non-cavitated or early carious lesions, thus reducing the caries incidence by preventing the progression of existing lesions or the development of new lesions [15]. It was confirmed that caries screening must monitor the lesion at its various dynamic stages including stages of mineral loss or gain to ensure successful caries management. Laser fluorescence technologies, using DIAGNOdent 2095 (KaVo, Biberach, Germany), relies on measuring the amount of fluorescence released by organic substances in dental tissues when stimulated by a 655 nm laser diode. Due to its increased transparency, light can travel through mature enamel without being deflected, while for affected enamel light will be scattered. DIAGNOdent could therefore offer a reliable and safe device, permitting the early diagnosis of non-cavitated lesions and detecting early changes in mineral content of enamel, as well as reflecting the remineralization potential of remineralizing agents [16–18].

Currently limited evidence exists regarding the remineralization potential of the newly introduced self-assembling peptide products. Some ex-vivo studies compared the impact of P11-4 to regular fluoride applications, demonstrating that this therapy could enhance remineralization and formation of de novo minerals in early enamel lesions. However, it was recommended to explore the clinical performance of such method and clarify any possible adverse effects [19]. Curodont™ Repair Fluoride Plus (vVARDIS Professional, Baar, Switzerland) was introduced to guide fluoride deposition into the depth of the enamel lesion. The peptide part of the product would self-assemble with the calcium and phosphorus from the saliva into a biomimetic matrix to enhance a guided enamel remineralization of the initial caries lesions and white spot lesions, representing a non-invasive method for enamel re-hardening and remineralization within few weeks following application [20]. The earlier version of Curodont was introduced in 2016 and proved clinical success and Curodont™ Repair Fluoride Plus was available in the market starting from 2019. Since then the available data was reported insufficiently and required additional investigation [21]. Given the current gap of knowledge [21], the current trial’s aim was to compare for the first time the remineralization potential of the self-assembling peptide P11-4 combined with fluoride to that of fluoride varnish using laser fluorescence as measurement device. The null hypothesis tested was that there will be no difference in the remineralization potential between self-assembling peptide (P11-4) combined with fluoride and fluoride varnish alone in the management of incipient carious lesions as assessed by laser fluorescence.

## Materials and methods

The current trial’s protocol was registered on www.ClinicalTrials.gov (NCT05094492). All procedures of the current randomized controlled clinical trial were conducted in agreement with the Helsinki declaration and its revision in 2013 and reported according to modified CONSORT guidelines [22]. Research Ethics Committee, Faculty of Dentistry at Cairo University reviewed and approved the protocol of the current trial (approval number:15|4|22). The current study is a randomized clinical trial, with parallel study design, 1:1 allocation ratio and superiority framework.

### Sample size calculation

Sample size was calculated using PS power and sample version 3.1.2 for windows. Based on a previous study [23], the DIAGNOdent reading within the sodium fluoride (NaF) group was normally distributed with standard deviation of 4.86. If the true difference between self-assembling peptide combined with fluoride and NaF alone means was 4, 24 teeth per group were deemed necessary to be able to reject the null hypothesis that the population means of the experimental and control groups are equal with a probability (power) of 0.8. This was increased to 29 subjects in each group to compensate for possible losses during follow-up. The Type I error probability associated with the testing of this null hypothesis was 0.05.

### Participants

Eligibility criteria:

a- Inclusion criteria:

- Age range between 18 and 25 years.
- Active non-cavitated incipient carious lesions on the facial smooth surface.
- DIAGNOdent score: 5–20.
- Patient with good compliance, who are willing to participate in the study.
- Controlled oral hygiene condition.

b- Exclusion criteria:

- Patients participating in another trial.
- Non-carious lesions.
- Cavitated carious lesions.
- Parafunctional habits.
- Pregnant females.

**Fig. 1:**
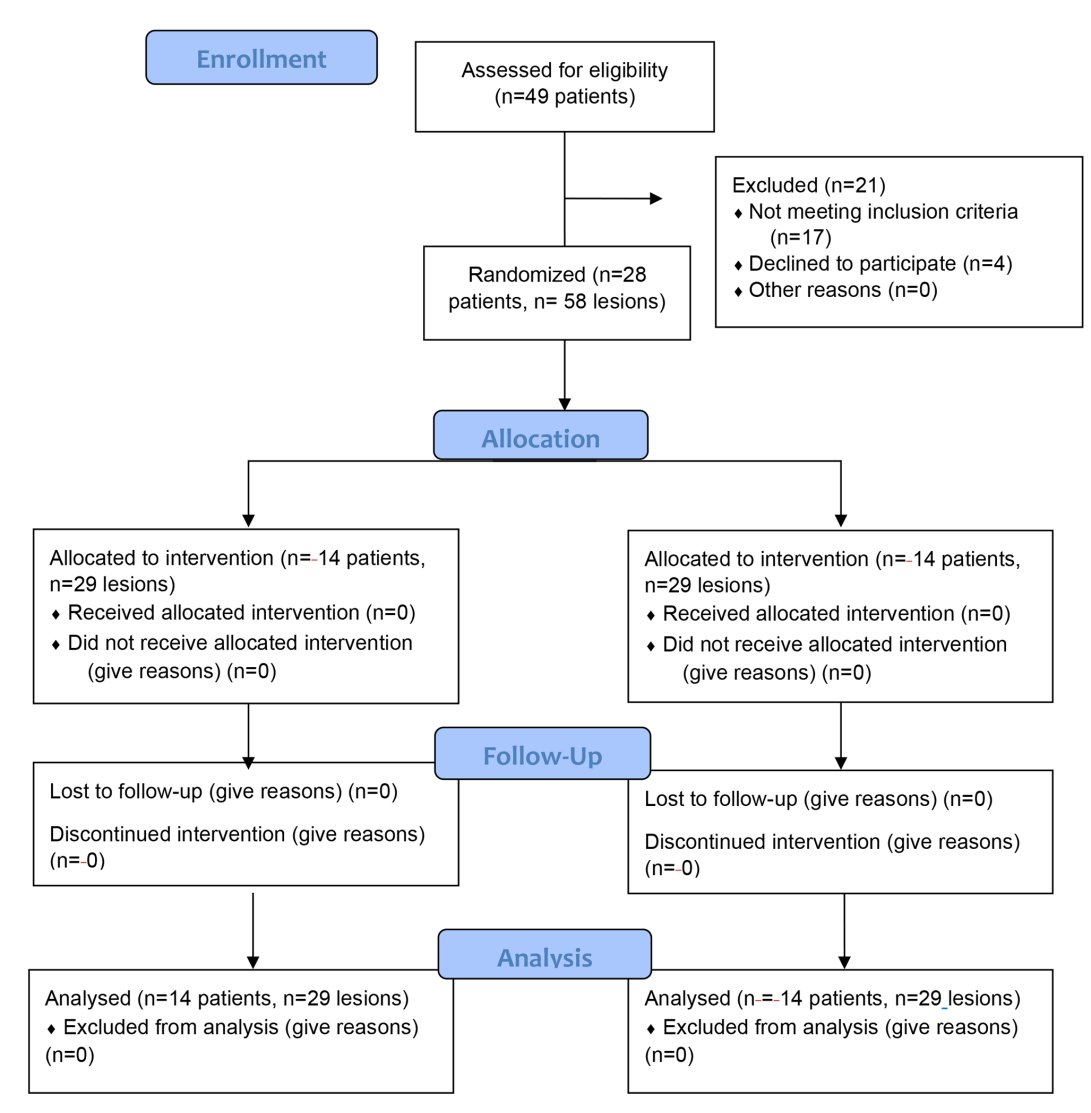
CONSORT flow diagram

Prior to examination, teeth were cleaned and polished and all patients were assessed under standardized operating conditions; pre-set dental unit position, operating light and air drying for approximately 5 s. A total of 79 participants were sampled, using convenient consecutive sampling, of which 58 participants met the eligibility criteria and informed consent was acquired from all eligible participants prior to enrollment in the current trial (Fig. 1 shows the flow of participants’ recruitment in the current trial).

### Randomization

Sequence generation was conducted by generating random numbers divided into two columns from 1 to 28 using www.randomization.com. Each participant picked his/her allocation from sequentially numbered opaque sealed envelopes, containing the generated random numbers. The current study was double blinded were participants and assessors were blinded to the materials’ assignment.

### Interventions

Prior to interventions application, a cheek retractor was applied, teeth surfaces were cleaned and polished using a cleaning and polishing brush (Pro-brush, Kerr, Orange, CA, USA). The involved teeth were isolated using cotton rolls and saliva ejector. All materials were applied according to their corresponding manufacturer’s instructions.

#### 1- Self-assembling peptide P11-4 with fluoride (Curodont™ Repair Fluoride Plus)

2% NaOCl solution was applied on the initial carious lesions for 20 s, then the solution was rinsed with water and the teeth were dried. The non-cavitated lesions were etched using Scotchbond™ Universal Etchant (3 M™ ESPE, St. Paul, Minnesota, USA) for 20 s to expose the subsurface lesion followed by rinsing with water for 20 s, followed by dryness using oil free air. Curodont Repair Fluoride Plus™ (vVARDIS Professional, Baar, Switzerland) was applied to the lesions and left for five minutes. Participants were then instructed not to rinse their mouth, eat or drink for the next 30 min.

#### 2–5% NaF Varnish (Bifluorid 10)

Fluoride varnish (Bifluorid 10, VOCO GmbH, Cuxhaven, Germany) was applied to the initial lesions without any surface pre-treatments using paint on brush technique. The varnish was left for 10–20 s on the lesion’s surface to be absorbed and dried afterwards using oil free air. Participants were then instructed not to brush their teeth for the next 12–24 h.

### Lesion’s assessment using laser fluorescence (DIAGNOdent)

Examiners were trained and calibrated for using DIAGNOdent 2095 (KaVo, Biberach, Germany) laser fluorescence device according to the device user manual. Inter-examiner agreement between assessors (OS and EA) showed an agreement score (kappa) of 0.84.

Calibration of the DIAGNOdent device was done using the ceramic standard provided with the device before usage on each patient, followed by obtaining zero baseline record through assessing fluorescence of a sound area on the labial surface of the teeth. After each ten readings the device was re-calibrated using the ceramic standard [24]. The tip of the DIAGNOdent was directed towards the initial carious lesions and rotated around a vertical axis. Three readings were acquired from each lesion and the highest reading was recorded. DIAGNOdent readings between 0 and 99 were categorized into four scores according to a previous classification, correlating the DIAGNOdent readings and the course of action based on lesion progression [25]. Score 1: 0–4 (healthy tooth structure), score 2: 5–10 (outer half enamel caries), score 3: 11–20 (inner half enamel caries) and score 4: 21+ (dentin caries). Lesions were assessed at baseline and after one, three and six months respectively. All lesions were examined by each observer separately without any communication using DIAGNOdent.

### Statistical analysis

Data was analyzed using Medcalc software, version 22 for windows (MedCalc Software Ltd, Ostend, Belgium). Data was explored for normality using Kolmogrov Smirnov test and Shapiro Wilk test. Continuous data showed normal distribution and were described using mean and standard deviation. Intergroup comparison between remineralizing agents was performed using independent t-test at each follow-up period (*p* < 0.05), while intragroup comparison between follow-up periods was performed using repeated measures ANOVA followed by Tukey-Kramer post-hoc test within each agent (Bonferroni corrected *p* < 0.008) and two-way ANOVA was used to test interaction of variables. Multiple regression was done to assess the relationship between DIAGNOdent readings, remineralizing agents and follow-up using simultaneous (enter) method. Comparison between categorical variables was performed using the chi-square test. Relative risk was used to evaluate the clinical significance.

## Results

### Demographic data

The present study was conducted on 28 participants with 58 incipient carious lesions that were randomly allocated to the intervention and the comparator arms (*n* = 29/group). After six months all participants were assessed with 100% retention rate. The trial included 10 males (35.7%) and 18 females (64.3%). Female patients’ percentage in the intervention group were higher than males; 10 females (71.4%) and 4 males (28.6%) respectively. In the comparator group there were 6 males (42.9%) and 8 females (57.1%), with no significant difference regarding gender distribution among groups (*p* = 0.4386). Mean age in the current study was 24.8 ± 4 years. In the intervention group mean age was 25.6 ± 4.8 years, while in the comparator group mean age was 24 ± 2.9 years, with no significant difference regarding age observed between the groups (*p* = 0.330). The included teeth were 35 incisors, 7 canines and 16 premolars in the current study (teeth distribution shown in Table 1), with no statistically significant difference regarding teeth distribution between groups (*p* = 0.1657).

**Table 1:** Teeth distribution among groups

Tooth type	Curodont Repair Fluoride Plus™	Bifluorid 10	Total
**Maxillary incisors**	10	17	**27**
**Mandibular incisors**	4	4	**8**
**Maxillary canines**	2	3	**5**
**Mandibular canines**	1	1	**2**
**Maxillary premolars**	5	0	**5**
**Mandibular premolars**	7	4	**11**
**Total**	**29**	**29**	**58**

### Evaluation of remineralization potential using DIAGNOdent

Between follow-ups, significantly lower intragroup DIAGNOdent readings were evident in Curodont Repair Fluoride Plus™ and NaF group independently (*p* < 0.0001). No significant differences were evident between both groups at baseline and after one month (*p* > 0.05), while at three- and six-months intergroup comparisons revealed significantly lower DIAGNOdent readings in the Curodont Repair Fluoride Plus™ group (*p* < 0.0001). The two-way ANOVA revealed statistically significant effect of material, follow-up and interaction of material and follow-up on DIAGNOdent readings (*p* < 0.001; Tables 2 and 3).

The multiple regression analysis demonstrated a coefficient of determination (R^2^) of 0.74, p‎<0.0001, with the beta coefficient (bi) being 1.12 (*p* = 0.0009) and − 3.75 (*p* < 0.0001) for the effect of remineralizing agent and follow-up respectively on the DIAGNOdent readings.

Relative risk was used to determine the effect size between both interventions, score 1 was considered negative (no risk of having caries), while scores 2 and 3 were considered positive (risk of having caries). There was a clinically significant difference between Curodont Repair Fluoride PlusTM and Bifluorid 10 after six months (RR = 0.4, 95% CI: 0.2382 to 0.6794; *p* = 0.0007).

**Table 2:** Mean and standard deviation of DIAGNOdent readings of both materials at each follow-up

Intervention Follow-up	Curodont Repair Fluoride Plus™		Bifluorid 10		*p* value
	Mean	SD	Mean	SD	
**Baseline**	15.93^a^	1.53	16.03^a^	1.47	0.7945
**1 month**	13.03^b^	2.04	12.72^b^	3.01	0.6478
**3 months**	7.45^c^	1.91	9.72^c^	1.70	< 0.0001*
**6 months**	3.62^d^	2.06	6.55^d^	2.02	< 0.0001*
**p value**	‎‎<0.0001‏‏*		< 0.0001‏‏*		

Means that do not share a letter vertically are significantly different. * corresponds to statistically significant difference

**Table 3:** Frequency and percentage of lesions scores at different follow-up periods for both materials based on DIAGNOdent scoring system

Follow-up	Curodont Repair Fluoride Plus™			Bifluorid 10			*p* value
	Score 1 (0–4)	Score 2 (5–10)	Score 3 (11–20)	Score 1 (0–4)	Score 2 (5–10)	Score 3 (11–20)	
**Baseline**	0 (0%)	0 (0%)	29 (100%)	0 (0%)	0 (0%)	29 (100%)	1.0000
**1 month**	0 (0%)	4 (13.8%)	25 (86.2%)	0 (0%)	3 (10.3%)	26 (89.7%)	0.6895
**3 months**	1 (3.4%)	26 (89.7%)	2 (6.9%)	0 (0%)	20 (69%)	9 (31%)	0.0126*
**6 months**	19 (65.5%)	10 (34.5%)	0 (0%)	4 (13.8%)	24 (82.8%)	1 (3.4%)	0.0001*
**p value**	< 0.0001*			< 0.0001*			

Score 1: 0–4 (healthy tooth structure), Score 2: 5–10 (Outer half enamel caries) and Score 3: 11–20 (Inner half enamel caries)

## Discussion

Early detection of incipient non-cavitated carious lesions requires instruments with high sensitivity and specificity [16]. Although many methods claimed to succeed in the detection of these incipient lesions, a huge discrepancy in the consistency of the acquired data was related to compromised specificity and/or sensitivity of the diagnostic method. On the other hand, the cost and ease of use favor the reliance on visual examination methods as a standard for the clinical assessment of the carious lesions [17, 18]. In this context, laser fluorescence diagnostic methods are currently considered to be reliable and efficient in early diagnosis of non-cavitated enamel carious lesions [15, 16, 26]. The DIAGNOdent device emits monochromatic light through its tip/sensor end to identify the lesion via the back scattered fluorescence, quantifying the lesion condition as well as its progression or arrest activity [27]. Despite its low specificity, it represents an efficient complementary device for detection of incipient smooth surface lesions comparable to photo documentation and ICDAS recordings [15, 28] with high reproducibility, especially when applied for smooth carious lesions than interproximal surfaces [29–31].

Early management of non-cavitated incipient carious lesions relied classically on topical fluoride applications, to produce harder apatite components on the enamel surface with greater resistance to acidic dissolution than natural hydroxyapatite [32]. The use of high concentrated fluoride gel products proved effective as a preventive measure as well as in the treatment of incipient carious lesions [33–36]. Yet, a phenomenon called lamination usually occurs, where the fluoride-induced remineralization process occurs at the surface, blocking the enamel pores, thus preventing the entrance of minerals into the deeper lesion’s body and inhibiting the full remineralization of the underlying tissues. An in-vitro study proved that cathodal iontophoresis (CIP) enhanced the remineralization potential of 5% NaF, where, the CIP created a significant rise in the calcium/phosphorus ratio and fluoride representing higher mineral density at the surface of the lesion [37].

Most novel remineralization agents’ formulations attempt to circumvent these restrictions by including additional potentially active components [38]. Such biomimetic material would form a scaffold within the incipient enamel lesion that could enhance further remineralization of the deeper layers by means of the salivary natural minerals [39]. In this context, self-assembled peptides (SAP) proved to restore damaged enamel and to enhance the development of de novo hydroxyapatite in the initial lesion [40]. The monomeric low viscosity peptide solution of the SAP would infiltrate the incipient carious lesions and promote the nucleation of the hydroxyapatite crystals, thus stopping further demineralization while promoting remineralization in deep subsurface lesions [10, 41].

A single time application of SAP on cervical carious lesions was able to minimize the size and halt the progression of the lesions with an improvement sustained for up to 180 days post-application [42]. Currently, of the three peptides known to promote early enamel caries remineralization, β-sheet forming self-assembling peptide (P11-4) is considered to present a great potential in these aspects. It represents a biomimetic agent that depends on the salivary natural dynamic remineralization mechanism, encouraging the formation of flower-like hydroxyapatite crystals for remineralization of the incipient enamel caries lesions [10, 43, 44]. Due to its low viscosity, P11-4 could speedily diffuse into the porosities of the incipient lesion, forming an elastomeric gel-like 3D fibrous matrix, which later promotes the biomineralization of the lesion [42]. P11-4 induced superior remineralization effects when compared to casein phosphopeptide amorphous calcium phosphate (CPP-ACFP) and NaF [45]. Furthermore, P11-4 was superior in treating incipient lesions existing on the buccal/labial surfaces of the young permanent dentition when compared to tricalcium phosphate fluoride [46]. Thus, a combination of P11-4 with fluoride could be supreme to using classical fluoride treatment method alone or even using previous generations of SAP solely [14].

A recent clinical trial demonstrated that previous generations of the material (Curodont Repair without fluoride) induced higher subsurface remineralization compared to fluoride varnish when applied to post orthodontic incipient lesions. Yet, both materials were unable to regress lesions beyond DIAGNOpen values of 5–10 (score 2) or ICDAS score of 1, indicating that both materials were successful only in partial healing of the enamel incipient lesions and their further progression [12]. A further clinical trial using Curodont Repair without fluoride revealed that 42.3% of lesions were totally or partially remineralized and that 34.6% of the lesions were arrested after six months [47]. Previously, P11-4 was combined with fluoride vanish application to treat incipient carious lesions, showing superior remineralization potential to the use of fluoride alone and preventing further lesion progression [14]. Adding fluoride to P11-4 in the current Curodont Repair Fluoride Plus™ formulation simplified this remineralization procedure, rather than using P11-4 followed by fluoride varnish in two separate steps.

In the current trial both tested materials, Curodont Repair Fluoride Plus™ and NaF, demonstrated comparable remineralization potential after one month, with superior results for the Curodont Repair Fluoride Plus™ at three and six months follow-ups. Results revealed a 60% less risk for caries progression for Curodont Repair Fluoride Plus™ compared to the NaF varnish after six months. Thus, the null hypothesis was rejected. According to the DIAGNOdent classification scores, NaF varnish was only able to change 13.8% of the lesions from score 3 to score 1, 82.8% of the lesions regressed from score 3 to score 2 and 3.4% of the lesions remained unchanged after six months. On the contrary, Curodont Repair Fluoride Plus™ showed complete healing of 65.5% of lesions to reach score 1, while 34.5% of the lesions demonstrated score 2, showing regression and remineralization, and 0% of the lesions were at score 3 after six months.

Despite the fact that 5% NaF was effective in rapid remineralization of early enamel lesions in-vitro when boosted with cathodal iontophoresis [37], it is worth mentioning that the effect of fluoride varnishes alone could require extended monitoring intervals when compared to some other remineralizing agents clinically [48]. Yet, SAP proved to be a safe and efficient method for enamel regeneration even amid a single application, enhancing mineral precipitates into the subsurface enamel structure [42]. In the current study, 100% of the lesions were totally (65.5%) or partially (34.5%) remineralized after six months in the Curodont™ Repair Fluoride Plus™ group. Its amalgamation with fluoride could have further augmented this remineralization potential. The multiple regression analysis revealed that 74% of the variations in the DIAGNOdent readings determining the remineralization potential can be explained by the changes in the type of the re-mineralizing agent and the follow up intervals. Beta coefficient (bi) was 1.12 (*p* = 0.0009) for effect of remineralizing agent on DIAGNOdent reading and − 3.75 (*p* < 0.0001) for effect of follow-up on DIAGNOdent reading. This demonstrates a strong significant correlation between the type of the remineralizing agent and its remineralization potential as demonstrated earlier by the Curodont Repair Fluoride Plus™ study group. Moreover, the regression analysis revealed a significant effect of the follow up intervals on the caries progress, where dental caries activity regressed all through the follow-up periods under the influence of both remineralizing agents.

Distribution of teeth type was not even between both groups, with more premolars in the Curodont Repair Fluoride Plus™ and more anterior teeth in Bifluorid 10 group. Yet this uneven distribution was not statistically significant. Moreover, the spearman’s correlation coefficient showed no or negligible correlation between tooth type and DIAGNOdent reading (rho=-0.0883, *p* = 0.5101), in agreement with a previous trial, demonstrating that the remineralization of incisors, canines and premolars in the cervical one third was nearly similar [49].

The application of the SAP could be considered a sophisticated and a multi-step technique when compared to the direct application of topical fluoride. Furthermore, the price and the economic value of using such a method as a preventive measure could be an obstacle and surely favors the use of simple and inexpensive topical fluoride agents. However, the need for SAP could be mandatory in some special cases, as the treatment of early enamel lesions and post-orthodontic white spot lesions (WSL), supported by the current evidence that the biomimetic remineralization of the SAP could be better than other conventional re-mineralizing agents. On the contrary, 5% NaF varnish was capable of re-mineralizing WSL both in-vitro and in-vivo, especially in patients with fair and poor oral hygiene [50, 51]. Moreover, a systematic review reported that NaF showed the best cumulative ranking in the prevention of incipient lesions in short term [52]. Despite lacking subsurface remineralization as explained earlier through the lamination phenomenon, traditional topical fluoride proved success and effectiveness according to a long history of clinical trials. Yet, it remains difficult to verify the actual beneficial value of the recent SAP due to the shortage of the reported long term clinical studies [33, 53].

Still, the current study’s results should be carefully interpreted in light of its limitations. First, the behavioral habits, oral hygiene routine and dietary habits were hard to unify during the whole follow up period for all the participants. A second limitation includes using DIAGNOdent as the only assessment method for the remineralization effect of both materials. DIAGNOdent indirectly assesses the remineralization potential of the agents through quantifying the lesion activity and bacterial byproducts. Low level of bacterial porphyrins in initial lesions could have limited the accuracy of DIAGNOdent, though the DIAGNOdent results were confirmed through matching to those reported by the clinical index (ICDAS-II) [15, 54, 55]. Third, a possibly longer follow-up period is required to accurately measure progression or regression of dental caries, especially after cessation of the re-mineralizing agents.

## Conclusions

The self-assembling peptide with fluoride showed superior remineralization potential to the conventional fluoride varnish alone for incipient carious lesions though demonstrating greater regression in the lesion’s activity when assessed using laser fluorescence. The combination of self-assembling peptide P11-4 and fluoride could offer a new tool in managing incipient carious lesions. Further controlled clinical trials are needed to assess the effect of repeated applications of such product over a prolonged period of time to verify its effectiveness and any possible adverse effects as a minimally invasive treatment modality for initial caries lesions.

## Data Availability

No datasets were generated or analysed during the current study.
